# Induction of MAPK- and ROS-dependent autophagy and apoptosis in gastric carcinoma by combination of romidepsin and bortezomib

**DOI:** 10.18632/oncotarget.6601

**Published:** 2015-12-14

**Authors:** Kwai Fung Hui, Po Ling Yeung, Alan K.S. Chiang

**Affiliations:** ^1^ Department of Paediatrics and Adolescent Medicine, Li Ka Shing Faculty of Medicine, The University of Hong Kong, Queen Mary Hospital, Pokfulam, Hong Kong, SAR, China

**Keywords:** bortezomib, romidepsin, gastric carcinoma, apoptosis, autophagy

## Abstract

Proteasome inhibitors and histone deacetylase (HDAC) inhibitors can synergistically induce apoptotic cell death in certain cancer cell types but their combinatorial effect on the induction of autophagy remains unknown. Here, we investigated the combinatorial effects of a proteasome inhibitor, bortezomib, and an HDAC inhibitor, romidepsin, on the induction of apoptotic and autophagic cell death in gastric carcinoma (GC) cells. Isobologram analysis showed that low nanomolar concentrations of bortezomib/romidepsin could synergistically induce killing of GC cells. The synergistic killing was due to the summative effect of caspase-dependent intrinsic apoptosis and caspase-independent autophagy. The autophagic cell death was dependent on the activation of MAPK family members (ERK1/2 and JNK), and generation of reactive oxygen species (ROS), but was independent of Epstein-Barr virus infection. *In vivo*, bortezomib/romidepsin also significantly induced apoptosis and autophagy in GC xenografts in nude mice. This is the first report demonstrating the potent effect of combination of HDAC and proteasome inhibitors on the induction of MAPK- and ROS-dependent autophagy in addition to caspase-dependent apoptosis in a cancer type.

## INTRODUCTION

Gastric carcinoma (GC) is the second leading cause of cancer-related death in the world. The mortality is especially high in Russia, Korea and Japan. Surgery is the only curative treatment for early stage of GC. However, GC remains asymptomatic for a long period of time and thus more than half of the cases are diagnosed clinically with distant metastasis. For such metastatic cases, the diseases are largely incurable and the 5-year survival rate is less than 10% [1]. Cisplatin and 5-fluorouracil are the most common types of chemotherapy for metastatic GC. The only available targeted therapy is trastuzumab but its application is limited to the human epidermal growth factor receptor 2-positive patients [2]. There is a clear need to explore more potential therapeutic agents for the treatment of GC.

Histone deacetylase (HDAC) inhibitors represent a novel class of therapeutic agents for cancer treatment. The mechanisms of actions of HDAC inhibitors include up-regulation of tumour suppressor genes, post-translational acetylation of histone and non-histone proteins and generation of reactive oxygen species (ROS) [3-5]. Activation of these mechanisms by HDAC inhibitors can lead to cell death through cell cycle arrest, senescence, apoptosis and autophagy [6-8]. Proteasome inhibitors are another emerging class of compounds for cancer treatment. It works by inhibiting the proteasomal degradation of ubiquitinated proteins which subsequently leads to apoptosis through induction of stress-related mechanisms in cancer cells [9, 10]. Recently, a couple of reports have shown that inhibition of proteasomal degradation by proteasome inhibitors can also induce autophagic cell death in cancer cells. For instance, a naturally occurring macrocyclic bisbibenzyl, Marchantin M, could non-covalently bind to the active sites of proteasome B1 and B5 subunits, and induce autophagic cell death in prostate cancer cells [11]. A FDA-approved proteasome inhibitor, bortezomib, was also reported to induce autophagic cell death in several cancer cell lines through activation of AMP-activated protein kinase, caspase-8 or jun N-terminal kinase (JNK) [12-14].

Our laboratory and others have reported that combination of proteasome and HDAC inhibitors can synergistically induce apoptosis of various types of cancer cells [15-20]. However, the combinatorial effect of proteasome and HDAC inhibitors on the induction of autophagy in cancer cells remains unexplored. GC cells were reported to be sensitive to both apoptotic and autophagic cell death [21, 22]. In this study, we aim to investigate the *in vitro* and *in vivo* effects of combining the FDA-approved proteasome inhibitor bortezomib with the FDA-approved HDAC inhibitor romidepsin on the induction of apoptosis and autophagy in GC cells. Our data showed that nanomolar concentrations of bortezomib/romidepsin could synergistically kill GC cells through the induction of apoptosis and autophagy. The autophagic cell death was dependent on ROS generation and the activation of ERK1/2 and JNK pathways but was independent of the presence of Epstein-Barr virus (EBV). Furthermore, bortezomib/romidepsin could also significantly induce apoptosis and autophagy and suppress the growth of GC xenografts in nude mice. This is the first study which demonstrates that bortezomib/romidepsin can induce concomitant apoptotic and autophagic cell death in GC cells and provides novel insight into the mechanism of synergistic action between proteasome and HDAC inhibitors on the induction of autophagy in cancer cells.

## RESULTS

### Combination of proteasome and HDAC inhibitors (i.e. bortezomib/romidepsin) synergistically inhibited proliferation of GC cells

**Figure 1:**
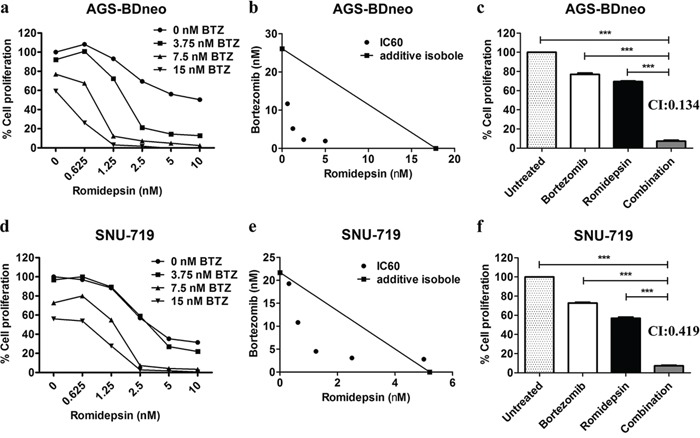
Effects of combination of bortezomib/romidepsin on cell proliferation of GC cells AGS-BDneo and SNU-719 cells were treated with various combinations of bortezomib/romidepsin for 48 hr. **A** & **D.** Data are presented as percentages of cell proliferation as determined by MTT assays. **B** & **E.** Synergisms of proliferation inhibition of the two cell lines were analyzed by isobologram analysis. **C** & **F.** Percentages of cell proliferation of GC cells upon treatment with combination of 7.5 nM bortezomib and 2.5 nM romidepsin were compared to those treated with either drug alone and combination indexes (CI) were calculated. Percentages of proliferating cells treated with bortezomib/romidepsin were compared with those untreated or treated with either drug alone using One-way ANOVA Dunnett's Multiple Comparison Test. P value < 0.05 was considered statistically significant (***p < 0.001). Error bars represent the standard error of mean (SEM) of data obtained in at least three independent experiments.

We tested whether the combination of bortezomib/romidepsin could induce synergistic killing of GC cells *in vitro*. AGS-BDneo and SNU-719 cells were treated with various combinations of bortezomib (0, 3.75, 7.5 and 15 nM) and romidepsin (0, 0.625, 1.25, 2.5, 5 and 10 nM) for 48 hr. The treated cells were assayed for percentage of cell proliferation by MTT assay. The drug combination yielded much stronger anti-proliferative effect when compared with either drug alone (Fig. 1a & 1d). The synergism between bortezomib/romidepsin was further analyzed by isobologram analysis (Fig. 1b & 1e). The isoboles lie to the left of the additive isoboles, indicating synergisms of bortezomib and romidepsin in their anti-proliferative effects. Combination of 7.5 nM bortezomib and 2.5 nM romidepsin induced significantly stronger killing of GC cells when compared with either drug alone (P<0.001; Fig. 1c & 1f). The combination indices of AGS-BDneo and SNU-719 cells treated with the combination of 7.5 nM bortezomib and 2.5 nM romidepsin are 0.134 and 0.419, respectively. The low combination indices (both <1) further demonstrated the strong synergistic anti-proliferative effects. The synergistic killing of GC cells could also be observed upon treatment with combinations of other HDAC and proteasome inhibitors (e.g. apicidin/bortezomib or romidepsin/carfilzomib combinations) (Supplementary Fig. 1). On the other hand, bortezomib/romidepsin did not induce synergistic killing of the normal human liver cell line MIHA, suggesting that the synergistic killing was specific to cancer cells (Supplementary Fig. 2).

### Bortezomib/romidepsin induced apoptosis of GC cells

**Figure 2:**
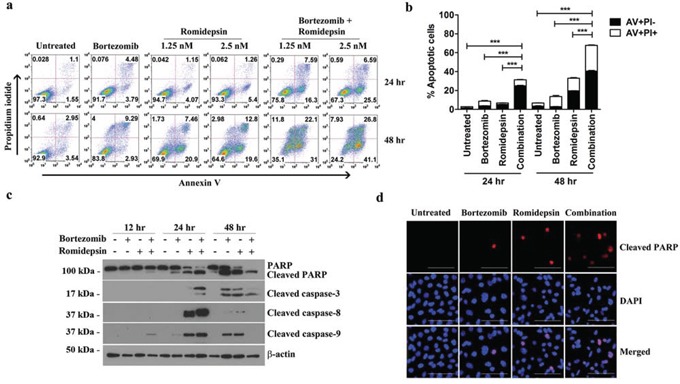
Effects of bortezomib/romidepsin on apoptosis of GC cells **A.** AGS-BDneo cells were treated with combination of 7.5 nM bortezomib and either 1.25 or 2.5 nM romidepsin or either drug alone for 24 hr and 48 hr, respectively. The treated cells were assayed for apoptosis by annexin V/propidium iodide (AV/PI) staining. **B.** Percentages of apoptotic cells treated with bortezomib/romidepsin were compared with those untreated or treated with either drug alone using One-way ANOVA Dunnett's Multiple Comparison Test. P value < 0.05 was considered statistically significant (***p < 0.001). **C.** AGS-BDneo cells were treated with combination of 7.5 nM bortezomib and 2.5 nM romidepsin or either drug alone for 12, 24 and 48 hr followed by detection of expression of PARP, cleaved PARP and cleaved caspase-3, -8 and -9 by western blot analysis. α-tubulin served as loading control. **D.** AGS-BDneo cells were treated with combination of 7.5 nM bortezomib and 2.5 nM romidepsin or either drug alone for 24 hr. Expression of cleaved PARP (red signals) was detected by immunofluorescent staining. DAPI (blue signals) stained the cell nuclei. Scale bar, 100 μm.

We then investigated whether the synergistic killing of GC cells by bortezomib/romidepsin was due to apoptosis. AGS-BDneo cells were treated with bortezomib, romidepsin or their combination for 24 and 48 hr. The treated cells were assayed for apoptosis by annexin V/propidium iodide (AV/PI) staining. Bortezomib/romidepsin treatment resulted in a higher percentage of apoptotic cells, when compared with either drug alone, in a time- and dose-dependent manner (Fig. 2a). At 48 hr, the percentages of AV-positive populations upon treatment with 7.5 nM bortezomib, 2.5 nM romidepsin and bortezomib/romidepsin increased to 12%, 32% and 68%, respectively (Fig. 2b). To further analyze the kinetics of apoptosis, we analyzed the proteolytic cleavage of PARP, caspase-3, -8 and -9 in AGS-BDneo cells by western blotting and immunofluorescent staining. Cleavage of PARP and caspases was observed at an earlier time point (∼12 hr) in the cells treated with bortezomib/romidepsin when compared with those treated with either drug alone (Fig. 2c & 2d). The results suggested that the synergistic killing of GC cells was related to the induction of apoptosis.

### The apoptosis induced by bortezomib/romidepsin was dependent on a reactive oxygen species (ROS)- and caspase-dependent intrinsic pathway

**Figure 3:**
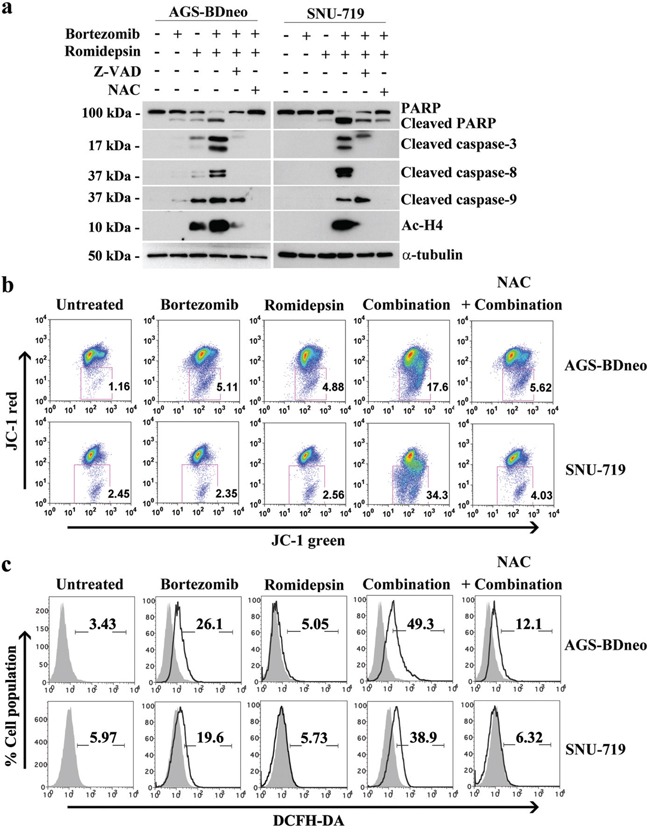
Roles of caspase activation and reactive oxygen species (ROS) generation in the apoptosis of GC cells induced by bortezomib/romidepsin **A.** AGS-BDneo and SNU-719 cells were pre-treated with either 50 μM Z-VAD-FMK or 12 mM N-acetyl-cystein (NAC) for 1 hr followed by treatment with combination of 7.5 nM bortezomib and 2.5 nM romidepsin or either drug alone for 24 hr. The treated cells were analyzed for the expression of PARP, cleaved PARP, cleaved caspase-3, -8 and -9 and acetylated histone H4 by western blot analysis. α-tubulin served as loading control. **B.** Decreases in mitochondrial membrane potential in GC cells upon treatment with bortezomib/romidepsin for 48 hr were analyzed by JC-1 assay. **C.** Generation of ROS was detected by DCFH-DA staining.

We have reported that the combination of HDAC and proteasome inhibitors could induce apoptosis of nasopharyngeal carcinoma (NPC) cells through an ROS- and caspase-dependent mechanism [19, 20]. Here, we tested whether the synergistic killing of GC cells by bortezomib/romidepsin was dependent on ROS generation and caspase activation. We treated GC cells with either NAC, an ROS scavenger, or Z-VAD-FMK, a pan-caspase inhibitor before treatment with bortezomib/romidepsin. We found that both NAC and Z-VAD-FMK could significantly reduce the cleavage of PARP and caspase-3, -8 and -9 (Fig. 3a). Since the intrinsic apoptotic initiator caspase-9 was activated, we also performed the JC-1 assay to investigate whether bortezomib/romidepsin could affect the mitochondrial membrane potential in GC cells. Our data showed that the drug combination could effectively reduce the mitochondrial membrane potential when compared with either drug alone (Fig. 3b). Moreover, we confirmed that bortezomib/romidepsin could also induce the generation of ROS in GC cells (Fig. 3c). Collectively, our data suggest that bortezomib/romidepsin could induce GC cell death through an ROS- and caspase-dependent intrinsic apoptotic pathway.

### Bortezomib/romidepsin also induced caspase-independent cell death

**Figure 4:**
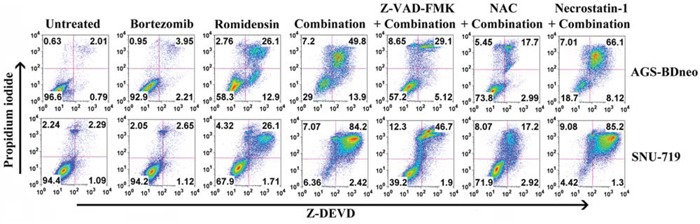
Percentages of caspase-dependent cell death induced by bortezomib/romidepsin in GC cells AGS-BDneo and SNU-719 cells were pre-treated with either 50 μM Z-VAD-FMK, 12 mM NAC or 50 μM necrostatin-1 for 1 hr followed by treatment with combination of 7.5 nM bortezomib and 2.5 nM romidepsin or either drug alone for 72 hr. Percentages of active caspase-3-positive/death cells was analyzed by Z-DEVD-FMK/PI staining.

It is reported that GC is subject to both apoptotic and autophagic cell death upon treatment with different therapeutic agents [21, 22]. We thus also investigated the mediation of other cell death through mechanisms in addition to apoptosis by the combination. According to the active caspase-3/PI assay, although caspase-3 activation (Z-DEVD +ve cells) was detected in the majority of dead cells (PI +ve cells), addition of the pan-caspase inhibitor Z-VAD-FMK could only partially reduce the percentage of dead cells induced by bortezomib/romidepsin (Fig. 4). Furthermore, addition of necrostatin-1, an inhibitor of necroptosis, did not suppress the cell death by bortezomib/romidepsin (Fig. 4). However, cell death was largely dependent on ROS generation because NAC significantly reduced the percentage of dead cells (Fig. 4). Taken together, our data indicated that a significant proportion of cells were killed through an ROS-dependent, but caspase-independent pathway.

### Bortezomib/romidepsin induced autophagic cell death

**Figure 5:**
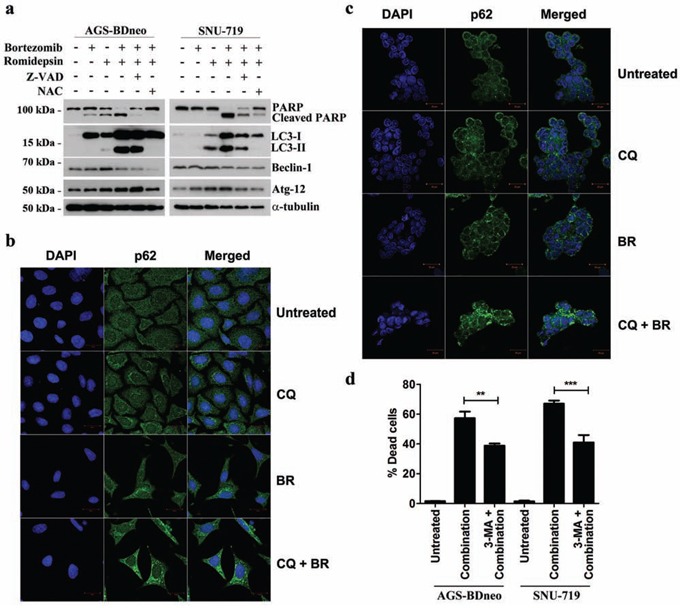
Effects of bortezomib/romidepsin on induction of autophagy in GC cells **A.** AGS-BDneo and SNU-719 cells pre-treated with either 50 μM Z-VAD-FMK or 12 mM NAC for 1 hr and then treated with bortezomib/romidepsin for 24 hr were analyzed for the expression of PARP, cleaved PARP, LC3-I/II, Beclin-1 and Atg-12. α-tubulin served as loading control. **B.** AGS-BDneo or **C.** SNU-719 cells were pre-treated with 10 μM chloroquine (CQ) for 1 hr followed by treatment with bortezomib/romidepsin (BR) for 24 hr. Expression of p62/SQSTM1 aggregates (green signal) was visualized with confocal microscopy. DAPI (blue signals) stained the cell nuclei. Scale bar, 20 μm. **D.** GC cells were pre-treated with 5 mM 3-MA for 1 hr followed by treatment with bortezomib/romidepsin (combination) for 72 hr. Percentages of death cells were detected by PI staining.

To investigate whether the caspase-independent cell death was mediated through autophagy, we examined the expression of several proteins known to be participated in the induction of autophagy, including LC3-I, LC3-II, Beclin-1 and ATG-12, in GC cells treated with bortezomib/romidepsin (Fig. 5a). The expression of LC3-I, LC3-II and Atg-12 was up-regulated upon treatment with bortezomib/romidepsin. The up-regulation of these proteins was significantly counteracted by the addition of NAC but not Z-VAD-FMK, indicating that the induction of autophagy was ROS-dependent but not caspase-dependent. To further confirm that bortezomib/romidepsin induced autophagy in GC cells, we detected the expression of p62/SQSTM1, an important marker of autophagy, in AGS-BDneo and SNU-719 cells upon treatment with bortezomib/romidepsin (BR) with or without chloroquine, an inhibitor of autophagic flux (Fig. 5b & 5c). We found that bortezomib/romidepsin induced the formation of p62/SQSTM1 protein aggregates in GC cells in the absence of chloroquine. The addition of chloroquine further increased the accumulation of the p62/SQSTM1 protein aggregates, substantiating the induction of autophagic machinery in GC cells. To examine the role of autophagy in the killing of GC cells, we treated both AGS-BDneo and SNU-719 cells with 3-MA, an inhibitor of autophagosome formation, before treatment with bortezomib/romidepsin and assayed for the percentage of cell death by PI staining (Fig. 5d). Our data showed that 3-MA could reduce ∼20% (similar to the percentages of cells resistant to the treatment with Z-VAD-FMK; refer to Fig. 4) of cell death mediated by bortezomib/romidepsin, suggesting that the killing of GC cells was also related to the induction of autophagy.

### The induction of autophagy by bortezomib/romidepsin was dependent on generation of ROS and activation of ERK1/2 and JNK pathways

**Figure 6:**
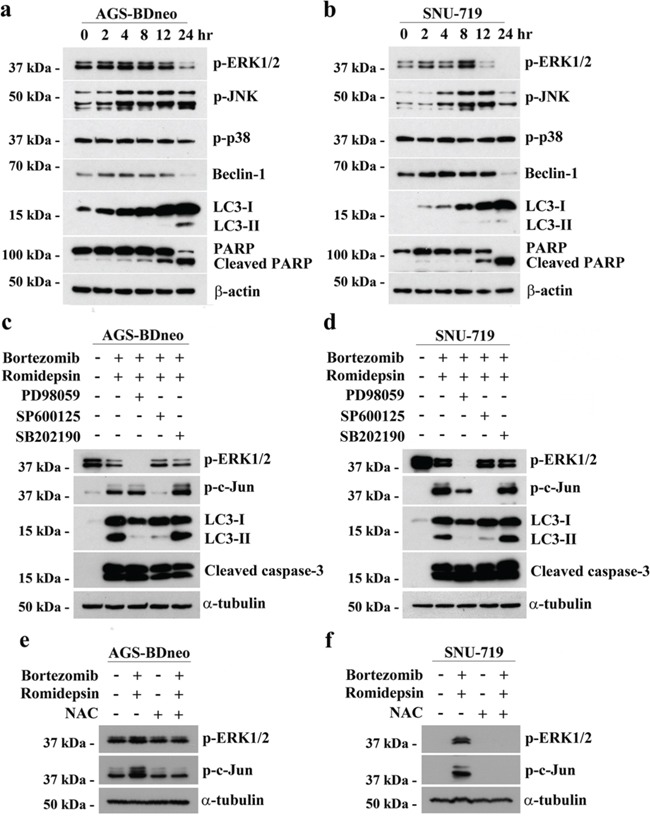
The involvement of MAPK pathways in the induction of apoptosis and autophagy by bortezomib/romidepsin **A.** AGS-BDneo and SNU-719 cells were treated with combination of 7.5 nM bortezomib and 2.5 nM romidepsin for 0, 2, 4, 8, 12 & 24 hr. The treated cells were analyzed for the expression of p-ERK1/2, p-JNK, p-p38, Beclin-1, LC3-I/II, PARP and cleaved PARP by western blot analysis. β-actin served as loading control. **B.** AGS-BDneo and SNU-719 cells were pre-treated with either 50 μM PD98059 (MEK inhibitor), 50 μM SP600125 (JNK inhibitor) and 20 μM SB202190 (p38 MAPK inhibitor) for 1 hr followed by treatment with combination of 7.5 nM bortezomib and 2.5 nM romidepsin or either drug alone for 24 hr. The treated cells were analyzed for the expression of p-ERK1/2, p-c-Jun, LC3-I/II and cleaved caspase-3 by western blot analysis. **C.** AGS-BDneo and SNU-719 cells were pre-treated with 12 mM NAC for 1 hr followed by treatment with combination of 7.5 nM bortezomib and 2.5 nM romidepsin or either drug alone for 8 hr. The expression of p-ERK1/2 and p-c-Jun was analyzed by western blot analysis. α-tubulin served as loading control.

The induction of apoptosis and autophagy in cancer cells are known to be regulated by several mitogen-activated protein kinases (MAPKs) pathways, including the extracellular signal-regulated kinase 1/2 (ERK1/2), c-Jun-NH_2_-terminal kinase (JNK) and p38 MAPK pathways [13, 23-25]. We, therefore, analyzed the expression kinetics of the phosphorylated forms of ERK1/2, JNK and p38 MAPK in GC cells before and after treatment with bortezomib/romidepsin. The phosphorylation of ERK1/2 and JNK was increased at around 2-4 hr post-treatment, followed by the cleavage of PARP and LC3 at 12 hr post-treatment. Phosphorylation of p38 was not affected by the drug combination (Fig. 6a & 6b). To further evaluate the roles of these pathways in the induction of autophagy and apoptosis by bortezomib/romidepsin, we investigated the cleavage of LC3 and caspase-3 in the GC cells following pharmacological inhibition of the MEK (upstream regulator of ERK1/2), JNK or p38 MAPK pathways. Our data showed that the MEK inhibitor PD98059 and JNK inhibitor SP600125 significantly reduced the cleavage of LC3-I to LC3-II but not the cleavage of caspase-3. However, the p38 MAPK inhibitor SB202190 did not affect the cleavage of both LC3 and caspase-3. As expected, phosphorylation of ERK1/2 and c-Jun, downstream signaling molecules of the MEK and JNK pathways was also significantly reduced by the MEK and JNK inhibitors (Fig. 6c & 6d). Consistently, pre-treatment with PD98059 and SP600125 also reduced the percentage of bortezomib/romidepsin-induced dead cells (Supplementary Fig. 3). Addition of NAC could significantly inhibit the phosphorylation of ERK1/2 and c-Jun, indicating that the activation of ERK1/2 and JNK pathways was downstream of ROS generation (Fig. 6e & 6f). Taken together, these data showed that autophagy induction by bortezomib/romidepsin was dependent on the generation of ROS and activation of ERK1/2 and JNK pathways.

### Induction of autophagy and apoptosis of GC by bortezomib/romidepsin was independent of the presence of Epstein-Barr virus (EBV)

Around 9% of GC is associated with EBV infection [26]. The expression of EBV latent and lytic proteins might affect the efficacy of the drug treatment [27-30]. We tested the effect of bortezomib/romidepsin on the parental EBV-negative AGS cells to examine whether there are any differential responses in the EBV-negative AGS cells when compared with the EBV-positive AGS-BDneo cells. We treated the AGS cells with bortezomib/romidepsin and analyzed the expression of PARP, cleaved caspase-3, cleaved caspase-9 and LC3-I/II in the cell lysates (Supplementary Fig. 4). Our data showed that the expression levels of both apoptotic and autophagic markers in the EBV-negative AGS cells were very similar to those observed in the EBV-positive AGS-BDneo cells upon treatment with bortezomib/romidepsin (refer to Fig. 3a & 5a). As the induction of EBV lytic cycle could lead to enhanced cell death in GC cells [31-33], we also analyzed the expression of EBV lytic proteins and replication of viral DNA upon treatment with bortezomib/romidepsin and found that bortezomib could suppress the lytic cycle induced by romidepsin in GC cells (Supplementary Fig. S5). Collectively, these data indicated that the synergistic killing of GC cells by bortezomib/romidepsin was likely independent of EBV.

### Bortezomib/romidepsin induced apoptosis and autophagy and suppressed GC tumour growth *in vivo*

**Figure 7:**
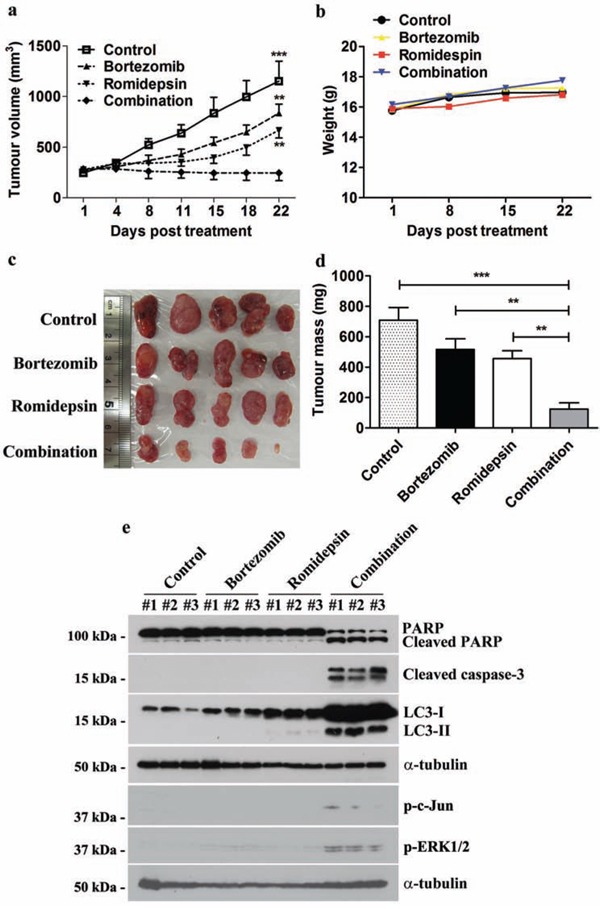
Effects of bortezomib/romidepsin on tumour growth suppression of GC xenografts in nude mice SNU-719 cells were subcutaneously injected into the right flanks of nude mice. When the tumours became palpable, the mice were either treated with 60 μg/kg bortezomib (day1-5 per week), 375 μg/kg romidepsin (day 1&4 per week) or their combination for 4 weeks by intraperitoneal injection. **A.** The size of tumours during the period of experiment was measured twice weekly using a caliper. Data are presented as the mean tumour volumes of mice in both treatment and control groups on the days post-treatment. **B.** The weight of nude mice was measured every week. **C.** Picture showing the size of tumours at the end of the experiments **D.** Average tumour masses of mice of control and treatment groups were shown. The tumour volumes and masses of mice treated with bortezomib/romidepsin were compared with those treated with vehicle control (DMSO) or either drug alone using One-way ANOVA Dunnett's Multiple Comparison Test. P value < 0.05 was considered statistically significant (***p < 0.001 and **p < 0.01). Error bars represent the standard error of mean (SEM) of tumour masses. **E.** Protein samples were extracted from the tumours and analyzed for the expression of PARP, cleaved PARP, cleaved caspase-3, LC3-I/II, p-c-Jun and p-ERK1/2 by western blotting. α-tubulin served as loading control.

We also evaluated the *in vivo* anti-tumour effect of bortezomib/romidepsin on GC xenografts established in nude mice. SNU-719 cells were inoculated subcutaneously at the right flanks of nude mice. The mice (n=5) were either treated with DMSO (vehicle control), 60 μg/kg bortezomib (day1-5 per week), 375 μg/kg romidepsin (day 1&4 per week) or their combination over 4 weeks by intraperitoneal injection. The growth of tumours and weight of mice were measured twice weekly during the experimental period. When compared with either bortezomib or romidepsin alone, administration of their combination resulted in much stronger tumour growth suppression but did not reduce the weight of the nude mice (Fig. 7a & 7b). On day 22, the average tumour mass in the control group increased to ∼700 mg. The average masses of tumours treated with either bortezomib or romidepsin alone increased to ∼500 mg and ∼450 mg, respectively, whilst those of the group treated with drug combination reduced to ∼100 mg (Fig. 7c & 7d). Furthermore, bortezomib/romidepsin also induced expression of cleaved PARP, cleaved caspase-3, LC3-I/II, p-c-Jun and p-ERK1/2 in the tumours resected from the nude mice (Fig. 7e). The data suggest that the *in vitro* effect of bortezomib/romidepsin on induction of apoptosis and autophagy could also be achieved *in vivo*.

## DISCUSSION

Our laboratory and others have reported that combination of proteasome and HDAC inhibitors can synergistically induce the killing of various types of cancer cell lines through the induction of apoptosis [15-20]. However, the combinatorial effect of proteasome and HDAC inhibitors on the induction of autophagy remains unexplored. GC cells are sensitive to both apoptotic and autophagic cell death [21, 22]. In this study, we demonstrated that combination of a proteasome inhibitor, bortezomib, and a HDAC inhibitor, romidepsin, could potently induce the killing of GC cells through a mechanism involving the summative effect of the caspase-dependent apoptosis and caspase-independent autophagy.

We analyzed the potential mechanisms of the induction of apoptosis and autophagy by combination of bortezomib/romidepsin in GC cells [13, 23-25]. We found that the majority of cells were killed by an intrinsic caspase-dependent apoptotic pathway because bortezomib/romidpesin induced the expression of active caspase-3 and reduced mitochondrial membrane potential in a significant proportion of cells (refer to Fig. 3b & Fig. 4). In addition to the induction of apoptosis, a population of cells was resistant to the caspase-dependent cell death but was killed through the induction of autophagic cell death (refer to Fig. 4 & Fig. 5). Both apoptosis and autophagy induced by bortezomib/romidepsin were dependent on ROS generation since the addition of NAC significantly reduced the generation of ROS and abrogated the cleavage of PARP, caspases and LC3. Prior to the induction of apoptosis and autophagy, the phosphorylated MAPKs (p-ERK1/2 and p-JNK), the apoptotic cascades (caspase-3, -8 and -9), and the molecules for autophagosome formation (Beclin-1 and LC3-I/II), were up-regulated. We further showed that the induction of autophagic cell death was dependent on the ERK1/2 and JNK pathways since inhibition of these two pathways by their specific blockers significantly reduced the expression of LC3-II and the percentage of dead cells. These findings are consistent with the reported roles of ERK1/2 and JNK pathways in the regulation of autophagic cell death in various types of cancer cells [23, 24]. Collectively, our data demonstrated that combination of proteasome and HDAC inhibitors (bortezomib/romidepsin) could induce concomitant apoptotic and autophagic cell death in GC cells through a mechanism involving the generation of ROS and activation of ERK1/2 and JNK pathways (refer to Supplementary Fig. 6).

Induction of autophagy can either be a pro-survival mechanism protecting cells against stress-induced killing [34-36] or a cell death mechanism induced by various anti-cancer agents [21, 22, 37-40]. Consistent with most of the reported data in other studies, our results showed that autophagic cell death induced by bortezomib/romidepsin was mediated through a strong production of ROS. We interpreted that the induction of autophagy will be cytoprotective when a moderate level of ROS is induced (e.g. under starvation). However, cell death mechanism would be activated if autophagy is induced through an acute increase of ROS in the cell. The disruption of lysosomes in cancer cells might be a possible mechanism of the autophagic cell death following strong production of ROS. It has been reported that acute generation of ROS can disrupt lysosomal membrane and lead to the leakage of hydrolytic enzymes which eventually cause cell death [41-43]. A recent report showed that induction of ROS generation by an anticancer agent, di-2-pyridylketone 4,4-dimethyl-3-thiosemicarbazone, could prevent the fusion of lysosomes with autophagosomes and eventually lead to induction of autophagic cell death [44]. In fact, HDAC and proteasome inhibitors had also been reported to disrupt the lysosomes in head and neck squamous cell carcinoma and pancreatic carcinoma cell lines [45, 46]. The data presented in this study might provide the ground for further in-depth investigation into the roles of ROS generation and lysosome disruption in the regulation of autophagic cell death.

Epstein-Barr virus (EBV) is closely associated with several types of malignancies, including endemic Burkitt lymphoma (BL), NPC and GC [47, 48]. In different EBV-associated malignancies, the virus expresses different latency patterns, namely type I, II, III or Wp-restricted latency [47, 48]. We have reported that combination of proteasome and HDAC inhibitors could synergistically inhibit the growth of Wp-restricted and latency III BL cells but not latency I BL cells [30]. These findings suggested that combination of proteasome and HDAC inhibitors could potentially target EBV in lymphoid cells of type III or Wp-restricted latency. However, we had shown that EBV-specific killing was not observed in latency II EBV-positive NPC cells by combination of proteasome and HDAC inhibitors [19, 20]. In the present study, we also compared the responses of the parental EBV-negative AGS and the EBV-positive AGS-BDneo GC cells, which exhibit EBV latency I pattern, to the treatment of bortezomib/romidepsin. Both EBV-negative and -positive GC cells were extremely sensitive to the induction of apoptosis and autophagy by bortezomib/romidepsin, indicating that the synergistic killing was not related to the presence of EBV.

Locoregional control by radical surgical resection is the only curative treatment for GC but more than half of GC cases have metastatic tumours at diagnosis [1]. The prognosis of such cases is poor because current chemotherapy has very limited effect on the disease [2]. Interestingly, we found that combination of bortezomib/romidepsin could induce apoptosis and autophagy of GC cells at concentrations (7.5 nM and 2.5 nM, respectively) much lower than the target therapeutic concentrations in patients' plasma (3400 nM and 790 nM, respectively) [49, 50]. We further demonstrated that bortezomib/romidepsin could effectively induce apoptosis and autophagy and suppress the growth of GC xenografts in nude mice, indicating that the *in vitro* synergistic action of bortezomib/romidepsin in GC cells could also be achieved *in vivo*.

In summary, bortezomib/romidepsin synergistically induced killing of GC cells *in vitro* and *in vivo*. The synergistic killing was due to the summative effect of caspase-dependent apoptosis and caspase-independent autophagy. The activation of ERK1/2 and JNK pathways, as well as the generation of ROS, were involved in the cell death mechanism. The research data support the testing of bortezomib/romidepsin as a potential therapeutic regimen for metastatic or recurrent GC and provide novel insight into the mechanism of synergistic action between proteasome and HDAC inhibitors in the induction of autophagic cell death of cancer cells.

## MATERIALS AND METHODS

### Cell lines and drug treatment

AGS and AGS-BDneo (gifts from Prof. L. Hutt-Fletcher, Louisiana State University, USA) are Epstein-Barr virus (EBV)-negative and recombinant EBV infected GC cell lines, respectively [51]. SNU-719 is a GC cell line containing native EBV genomes (purchased from the Korean Cell Line Bank) [52, 53]. To assay for cell death, GC cells grown to 70% confluence were treated with various combinations of bortezomib or carfilzomib (Selleck Chemicals, Houston, TX) and apicidin or romidepsin (Selleck Chemicals) for 24 or 48 hr. To inhibit ROS generation, caspase activation and autophagosome formation, cells were pre-treated with either 12 mM N-acetyl-cysteine (NAC; Sigma-Aldrich, St. Louis, MO), 50 μM Z-VAD-FMK (Torcris Bioscience, Bristol, UK), 50 μM necrostatin-1 or 5 mM 3-Methyladenine (Sigma-Aldrich), respectively, for 1 hr before treatment with bortezomib/romidepsin. The MEK inhibitor PD98059, JNK inhibitor SP600125 and p38 MAPK inhibitor SB202190 were purchased from Calbiochem (San Diego, CA). AGS and AGS-BDneo cell lines were authenticated with an AmpF/STR Identifiler PCR Amplication Kit (Applied Biosystems, Foster City, CA), according to the manufacturer's protocol in August 2011. The data were analyzed by GeneScan and GeneMapper™ ID Software (Applied Biosystems). The STR profiles were compared with DSMZ database. SNU-719 cell line was authenticated by the Korean Cell Line Bank.

### MTT assay

GC cells were seeded in triplicates in 96-well plates and treated with various combinations of either bortezomib (0, 3.75, 7.5 and 15 nM) or carfilzomib (0, 6.25, 12.5 and 25 μM) and either romidepsin (0, 0.625, 1.25, 2.5, 5 and 10 nM) or apicidin (0, 0.16, 0.3, 0.625, 1.25 and 2.5 μM) for 48 hr. 3-(4,5-Dimethylthiazol-2-yl)-2,5-diphenyltetrazolium bromide (MTT; Invitrogen, Carlsbad, CA) assay was performed and percentage of cell proliferation was calculated as previously described [20, 28, 29].

### Annexin V/propidium iodide (AV/PI) assay

GC cells were incubated with drugs for 24 or 48 hr. Following the incubation, both floating and adherent cells were collected and washed once with phosphate buffered saline (PBS). Cells were stained with FITC-AV and PI (BD Pharmingen™, Heidelberg, Germany) and the percentage of AV/PI-positive cells was calculated as previously described [20, 28].

### Immunocytochemistry

GC cells grown on cover slips coated with 0.1% gelatin were treated with drugs for 24 hr. Cells were fixed with acetone for 10 minutes at room temperature. The fixed cells were then stained with cleaved poly (ADP-ribose) polymerase (PARP) or p62/SQSTM1 rabbit polyclonal antibody (1:200; Cell Signaling Technology, Beverly, MA) overnight at 4°C. Expression of proteins was visualized with Alexa Fluor 488 F(ab')2 fragment of goat anti-rabbit IgG antibody (1:500; Invitrogen) under fluorescent microscope or Carl Zeiss LSM 710 confocal microscope (Carl Zeiss, Germany). Nuclei of cells were stained with 4′,6-diamidino-2-phenylindole (DAPI) (Roche, Mannheim, Germany).

### Western blot analysis

GC cells were treated with drugs for 12, 24 or 48 hr. Proteins from the cell cultures were extracted and western blot analysis was performed as described previously [29]. Apoptotic proteins were detected with the antibodies reported previously [28]. Histone acetylation was detected with acetyl-histone H4 rabbit polyclonal antibodies (1:2000; Millipore, Temecula, CA). Expression of autophagic proteins was detected with Beclin-1, Atg12 and LC3-I/II rabbit polyclonal antibodies, respectively (1:1000; Cell Signaling Technology). Expression of phosphorylated JNK1/2 isoforms, phosphorylated c-Jun, phosphorylated ERK1/2 and phosphorylated p38 was detected with p-JNK, p-c-Jun, p-ERK1/2 and p-p38 rabbit polyclonal antibodies, respectively (1:1000; Cell Signaling Technology). Expression of human α-tubulin was detected with α-tubulin antibody (1:5000; Sigma-Aldrich) as a loading control.

### JC-1 assay

GC cells were treated with drugs for 48 hr. One million cells were collected and washed once with PBS. The cells with loss of mitochondrial membrane potential, which was reflected by decreased JC-1 red signal, were detected with Flow Cytometry Mitochondrial Membrane Potential Detection Kit (BD Biosciences) following the manufacturer's instructions. The stained cells were detected by flow cytometry (LSRII, BD Biosciences). Data were analyzed by FlowJo software (Tree Star).

### Dichlorofluoescein diacetate (DCFH-DA) assay

DCFH-DA (Sigma-Aldrich) assay was performed to analyze the intracellular reactive oxygen species (ROS) level. AGS-BDneo and SNU-719 cells were treated with bortezomib/romidepsin for 24 hr. The adherent cells were then stained with 2 μM DCFH-DA. The change of ROS level was measured by flow cytometry as previously described [20].

### Active caspase-3 assay

GC cells were treated with drugs for 72 hr. The treated cells were collected and washed once with PBS. Activation of caspase (DEVD) and cell death (PI) were detected with CaspaTag Caspase-3, -7 In Situ Assay Kit, Fluorescein (Millipore, Billerica, MA) following the manufacturer's instructions. The stained cells were detected by flow cytometry (LSRII, BD Biosciences). Data were analyzed by FlowJo software (Tree Star).

### Nude mice experiment

Female BALB/c nude (nu/nu) mice were purchased at 5-6 weeks of age from the Laboratory Animal Unit, The University of Hong Kong. SNU-719 (1 × 10^7^) cells were re-suspended in 200 μl serum-free culture medium. The cells were subcutaneously injected at the right flanks of nude mice at 6-7 weeks of age. When the tumours became palpable, 60 μg/kg body weight of mice of bortezomib (day1-5 per week), 375 μg/kg romidepsin (day 1&4 per week) or their combination dissolved in DMSO in 10 μl was administered to the nude mice of the treatment groups (n=5) by intraperitoneal (IP) injection for 4 weeks. Equal volume of DMSO was administered to the nude mice of the control group (n=5). The size and weight of the tumours were measured as described previously [20, 28].

### Quantitative PCR assay

AGS-BDneo cells were treated with bortezomib/romidepsin for 72 hr. The EBV viral load was analyzed by quantitative PCR as described previously [29]. Data were determined by triplicate wells in a 96-well plate format.

### Statistical analysis

*In vitro* experiments were performed in triplicate and repeated at least 3 times. Data were analyzed for statistical significance using One-way ANOVA Dunnett's Multiple Comparison Test. P value < 0.05 was considered statistically significant. Synergism of bortezomib and romidepsin was analyzed with isobologram analysis and combination index (CI) calculation as described previously [20]. In the isobologram, the curves that lie under the additive isobole suggest synergism and vice versa [54]. The CI was calculated using the Chou and Talalay method using Microsoft Excel software [55]. CI<1, =1 and >1 represent synergy, additive and antagonism, respectively. All statistical analyses were performed with GraphPad Prism Version 5.0 software.

## SUPPLEMENTARY FIGURES


