# Supplementary material for: Induction of MAPK- and ROS-dependent autophagy and apoptosis in gastric carcinoma by combination of romidepsin and bortezomib
Source: Oncotarget. 2015 Dec 14;7(4):4454–67. doi: 10.18632/oncotarget.6601 (PMC4826218; doi:10.18632/oncotarget.6601)
Supplement: Supplementary file 1 [file oncotarget-07-4454-s001.pdf]

SUPPLEMENTARY FIGURES

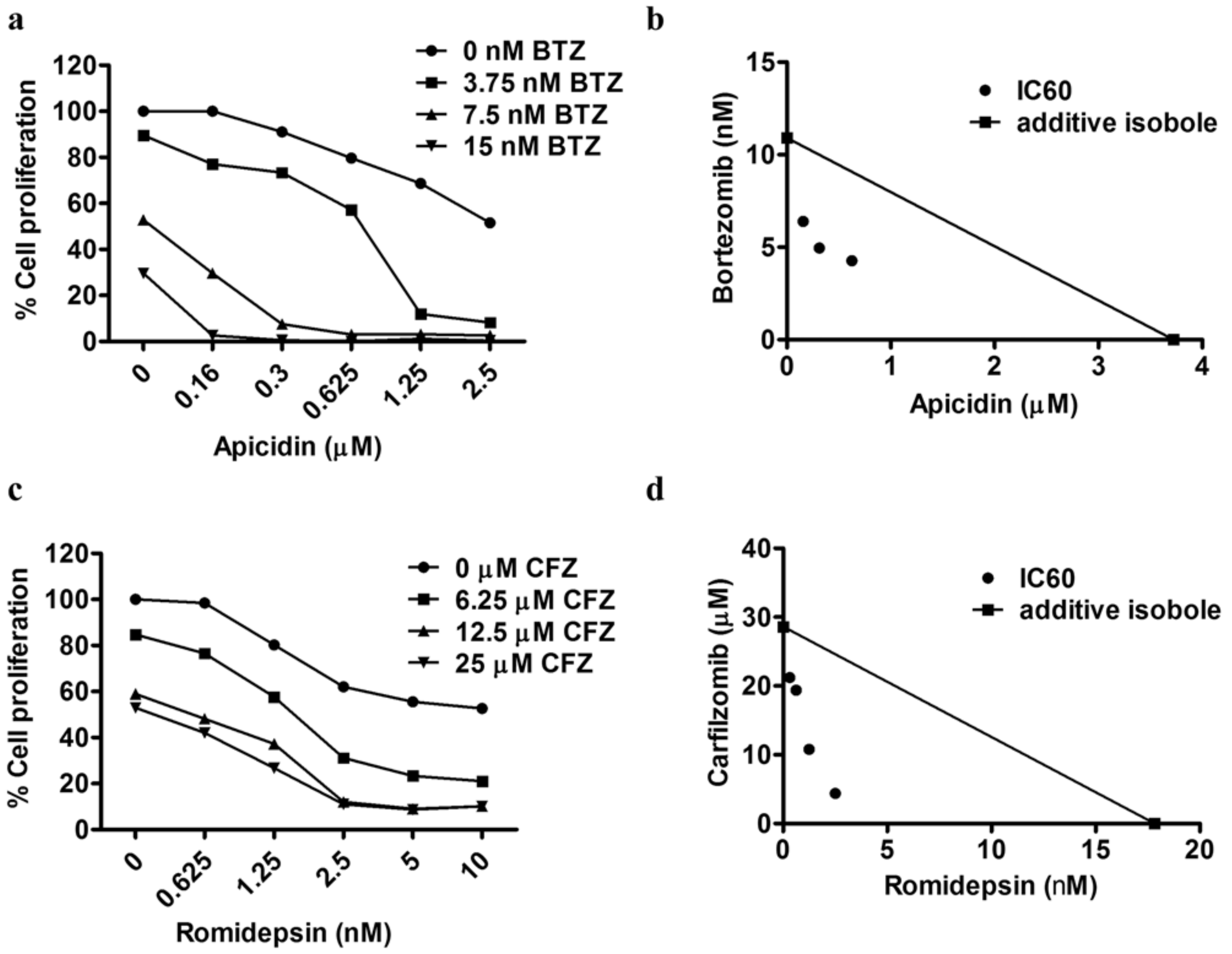

Supplementary Figure S1: Effect of combination of bortezomib/apicidin or carfilzomib/romidepsin on cell proliferation of GC cells. AGS-BDneo cells were treated with various combinations of bortezomib/apicidin or carfilzomib/romidepsin for 48 hr. **A & C.** Data are presented as percentages of cell proliferation as determined by MTT assays. **B & D.** Synergisms of proliferation inhibition of the two cell lines were analyzed by isobologram analysis.

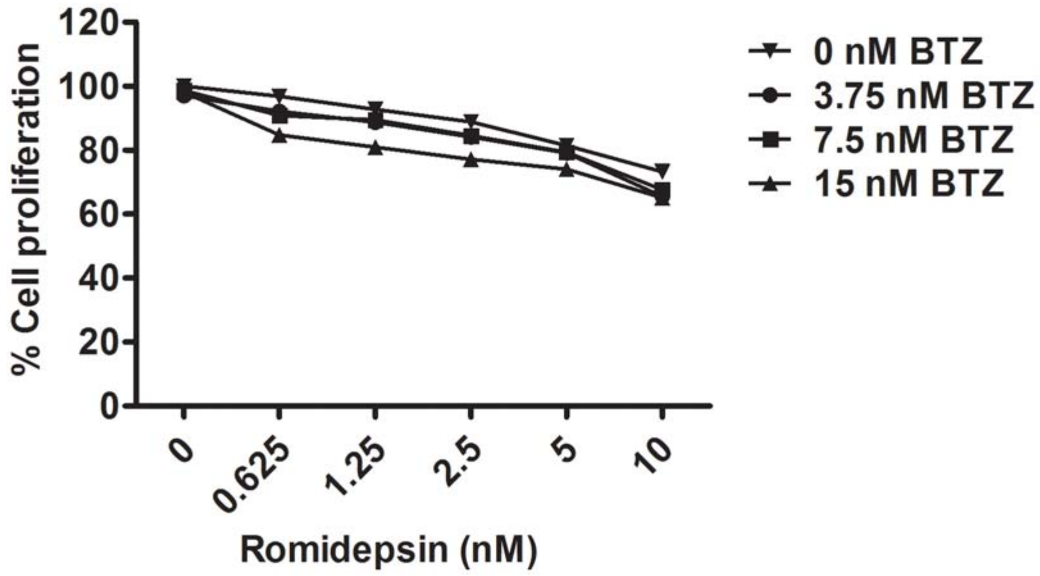

Supplementary Figure S2: Effect of combination of bortezomib/romidepsin on cell proliferation of normal liver cells. MIHA cells were treated with various combinations of bortezomib/romidepsin for 48 hr. Data are presented as percentages of cell proliferation as determined by MTT assays.

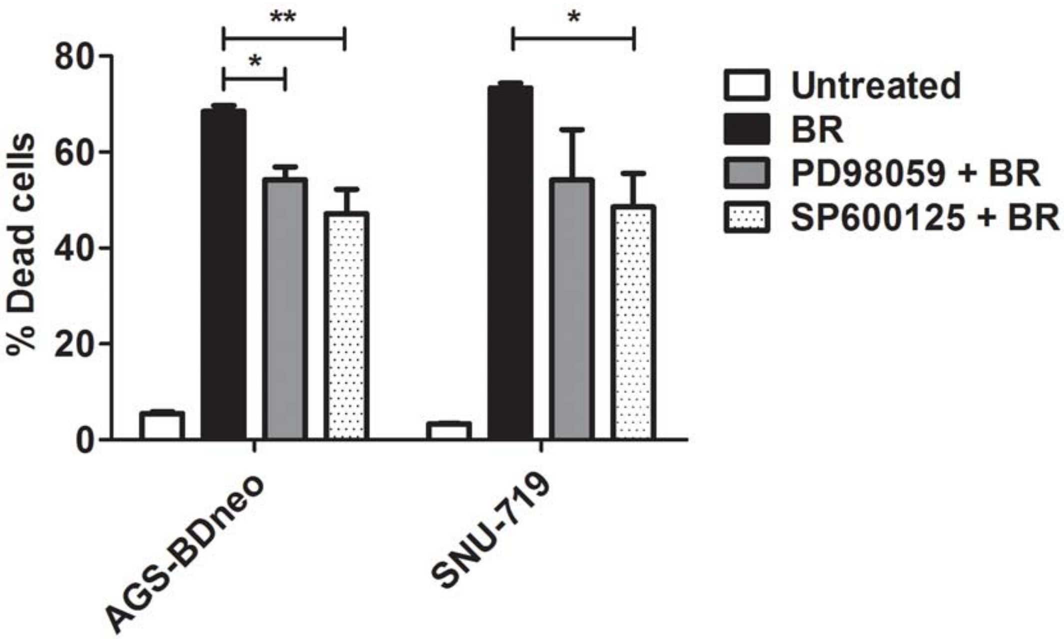

Supplementary Figure S3: Effects of MEK and JNK inhibitors on bortezomib/romidepsin induced GC cell death. AGS-BDneo and SNU-719 cells were pre-treated with either 50 µM PD98059 (MEK inhibitor) or 50 µM SP600125 (JNK inhibitor) for 1 hr followed by treatment with combination of 7.5 nM bortezomib and 2.5 nM romidepsin for 72 hr. Percentages of death cells were detected by PI staining.

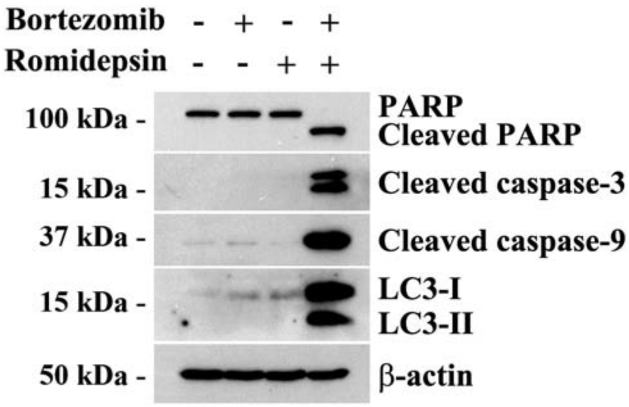

Supplementary Figure S4: Roles of EBV in the autophagy and apoptosis induced by bortezomib/romidepsin. EBV-negative AGS cells were treated with combination of 7.5 nM bortezomib and 2.5 nM romidepsin or either drug alone for 24 hr. The treated cells were analyzed for the expression of PARP, cleaved caspase-3 and LC3-I/II by western blot analysis. β-actin served as loading control.

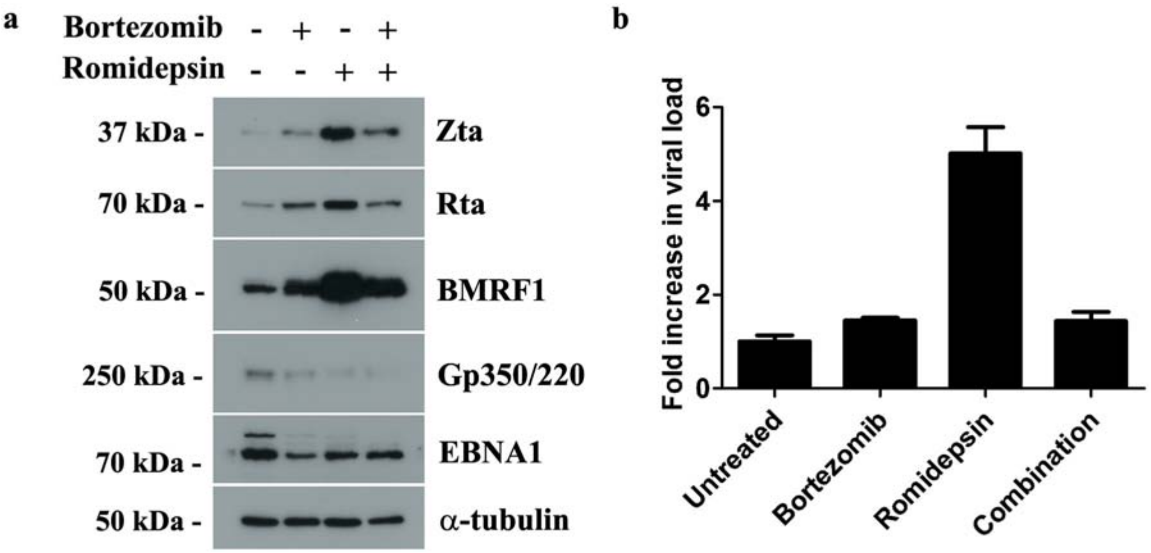

Supplementary Figure S5: Effect of bortezomib/romidepin on reactivation of EBV lytic cycle in GC cells. AGS-BDneo cells were treated with combination of 7.5 nM bortezomib and 2.5 nM romidepsin or either drug alone for 48 or 72 hr. **(A)** At 48 hr, expression of the EBV immediate-early (Zta and Rta), early (BMRF1) and late (gp350/220) lytic proteins and EBV latent protein (EBNA1) was analyzed by western blotting. α-tubulin was detected as a loading control. **(B)** At 72 hr, quantitative PCR was performed to analyze the induction of EBV DNA replication in AGS-BDneo cells. Data are presented as fold increase in viral genomes/cell in the treated cells compare with the untreated control.

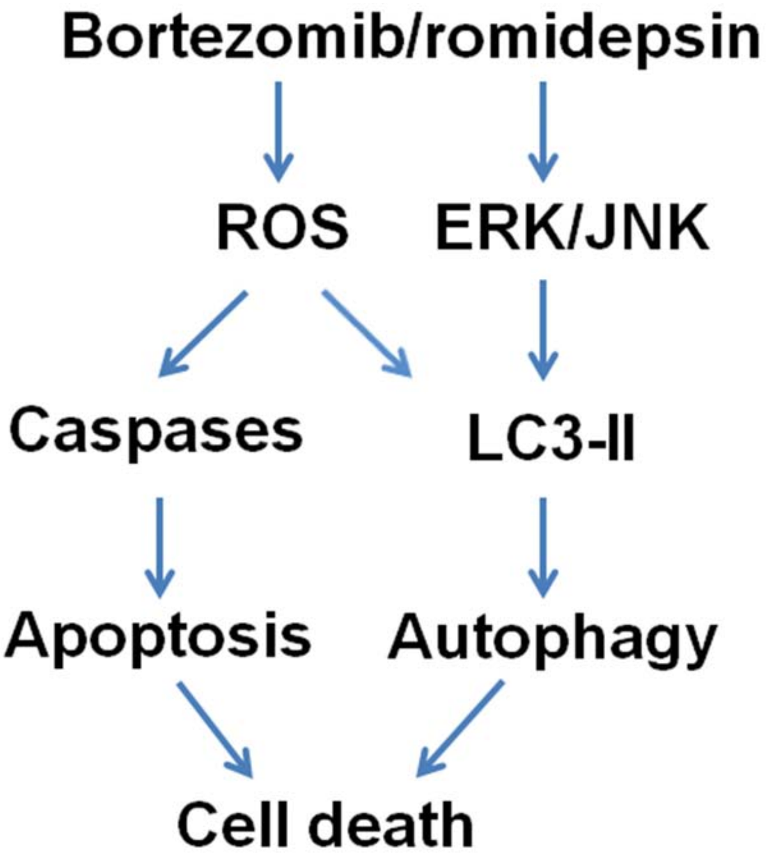

Supplementary Figure S6: Schematic diagram showing the mechanisms of induction of autophagy and apoptosis in GC cells by bortezomib/romidepsin.
